# The Effect of Processing and Seasonality on the Iodine and Selenium Concentration of Cow’s Milk Produced in Northern Ireland (NI): Implications for Population Dietary Intake

**DOI:** 10.3390/nu10030287

**Published:** 2018-03-01

**Authors:** S. Maria O’Kane, L. Kirsty Pourshahidi, Maria S. Mulhern, Ruth R. Weir, Sarah Hill, Jennifer O’Reilly, Diana Kmiotek, Christian Deitrich, Emer M. Mackle, Edel Fitzgerald, Carole Lowis, Mike Johnston, J.J. Strain, Alison J. Yeates

**Affiliations:** 1 Nutrition Innovation Centre for Food and Health (NICHE), University of Ulster, Cromore Road, Coleraine BT52 1SA, UK; mariaokane29@outlook.com (S.M.O.); k.pourshahidi@ulster.ac.uk (L.K.P.); m.mulhern@ulster.ac.uk (M.S.M.); rrweir89@gmail.com (R.R.W.); e_mackle@outlook.com (E.M.M.); fitzgerald-e3@ulster.ac.uk (E.F.); jj.strain@ulster.ac.uk (J.J.S.); 2 LGC, Queens Road, Teddington, Middlesex TW11 0LY, UK; Sarah.Hill@lgcgroup.com (S.H.); Jennifer.Oreilly@lgcgroup.com (J.O.); Dkmiotek@hotmail.co.uk (D.K.); Christian.Deitrich@lgcgroup.com (C.D.); 3 The Dairy Council for Northern Ireland, Edgewater Road, Belfast BT3 9JQ, UK; carole@foodcommunications.co.uk (C.L.); mjohnston@dairyuk.org (M.J.)

**Keywords:** iodine, selenium, cow’s milk, seasonality, processing, dietary intake, public health

## Abstract

Cow’s milk is the most important dietary source of iodine in the UK and Ireland, and also contributes to dietary selenium intakes. The aim of this study was to investigate the effect of season, milk fat class (whole; semi-skimmed; skimmed) and pasteurisation on iodine and selenium concentrations in Northern Ireland (NI) milk, and to estimate the contribution of this milk to consumer iodine and selenium intakes. Milk samples (unpasteurised, whole, semi-skimmed and skimmed) were collected weekly from two large NI creameries between May 2013 and April 2014 and were analysed by inductively coupled plasma-mass spectrometry (ICP-MS). Using milk consumption data from the National Diet and Nutrition Survey (NDNS) Rolling Programme, the contribution of milk (at iodine and selenium concentrations measured in the present study) to UK dietary intakes was estimated. The mean ± standard deviation (SD) iodine concentration of milk was 475.9 ± 63.5 µg/kg and the mean selenium concentration of milk was 17.8 ± 2.7 µg/kg. Season had an important determining effect on the iodine, but not the selenium, content of cow’s milk, where iodine concentrations were highest in milk produced in spring compared to autumn months (534.3 ± 53.7 vs. 433.6 ± 57.8 µg/kg, respectively; *p* = 0.001). The measured iodine and selenium concentrations of NI milk were higher than those listed in current UK Food Composition Databases (Food Standards Agency (FSA) (2002); FSA (2015)). The dietary modelling analysis confirmed that milk makes an important contribution to iodine and selenium intakes. This contribution may be higher than previously estimated if iodine and selenium (+25.0 and +1.1 µg/day respectively) concentrations measured in the present study were replicable across the UK at the current level of milk consumption. Iodine intakes were theoretically shown to vary by season concurrent with the seasonal variation in NI milk iodine concentrations. Routine monitoring of milk iodine concentrations is required and efforts should be made to understand reasons for fluctuations in milk iodine concentrations, in order to realise the nutritional impact to consumers.

## 1. Introduction

Iodine and selenium are essential trace elements required for the maintenance of thyroid hormone homeostasis [1]. Selenium is necessary for immune, brain and muscle function and is essential for reproductive function [2,3,4]. Iodine is required for the production of thyroid hormones, triiodothyronine (T3) and thyroxine (T4) that are essential during pregnancy and infancy for brain and neurological development [5,6]. Iodine deficiency is recognised to be the most preventable cause of mental impairment and is a major public health concern worldwide [7]. The UK and Ireland were historically considered to be iodine sufficient [8], but emerging evidence shows that the UK and Ireland are mildly iodine deficient [9,10,11,12]. Salt iodisation has been the primary approach worldwide to control and prevent iodine deficiency and monitoring of salt iodisation programmes has demonstrated this to be an effective strategy [13,14,15]. In some European countries including the UK, policy makers have been reluctant to implement a salt iodisation programme as this may conflict with public health efforts to reduce salt intake for the prevention of cardiovascular disease [14]. With the growing problem of iodine deficiency and the lack of prophylaxis with iodised salt, alternative strategies are required to increase iodine status.

In many countries which have not implemented a mandatory iodised salt programme, including the UK, milk is the most important dietary source of iodine [16,17,18,19,20,21,22]. For the UK adult (19–64 years) population, milk and dairy products typically contribute 35% to daily iodine intake and 6% to dietary selenium intake [12]. Estimated nutrient intakes are based on food composition data [23] which may not provide an accurate estimate of iodine and selenium intake, as iodine and selenium concentrations have not been systematically updated for many years [24,25]. Variations in the micronutrient content of similar foods and changes in nutritional composition over time can also limit the accuracy of food composition data used to estimate dietary intakes [26]. 

The iodine and selenium concentrations of milk are highly variable and, considering the importance of milk to iodine and selenium intakes in the UK and Ireland, a greater understanding of the factors affecting the iodine and selenium concentration in milk is essential. The iodine concentrations in milk are directly proportional to the iodine concentrations in feedstuffs which are routinely used in farming practices for animal health [27,28]. Moreover, previous research has demonstrated that season of milk production, fat content of milk, milk pasteurisation and organic farming can significantly affect the iodine and selenium concentration of milk [29,30,31,32,33,34,35]. Dairy management systems often vary across countries and can also affect the iodine and selenium concentrations of milk [36]. 

A comprehensive analysis of the iodine and selenium concentrations in Northern Ireland (NI) produced cow’s milk and the factors affecting iodine and selenium concentrations in NI milk is warranted given the importance of milk to dietary iodine intake. Accordingly, a study of retail milk produced in NI was undertaken to quantify the iodine and selenium concentrations in NI retail milk and to assess the effect of milk fat class, pasteurisation and season on concentrations. An additional aim of this study was to estimate the contribution of milk to iodine and selenium intakes, using measured NI iodine and selenium concentrations, at current UK population milk intakes.

## 2. Materials and Methods

### 2.1. Milk Sampling

The sampling method used was designed to be representative of cow’s milk sold at retail in NI. Milk samples were collected from May 2013 to April 2014 on a weekly basis from two of the largest creameries in NI. Samples (1 L) of skimmed, semi-skimmed, whole and unpasteurised milk were obtained on each occasion. A total of 376 samples were collected, kept chilled and transferred to Ulster University Coleraine where they were stored at −20 °C prior to analysis. Weekly samples from the two creameries were then pooled to create monthly samples of each milk type (skimmed, semi-skimmed and whole). The monthly milk samples of skimmed (*n* = 12), semi-skimmed (*n* = 12), whole milk (*n* = 12) and unpasteurised (*n* = 12) were then grouped together to create seasonal samples (Spring: March–May; Summer: June–August; Autumn: September–November; Winter: December–February). To investigate the differences in iodine and selenium concentrations between winter and summer milk as described by others [37], winter/indoor period was defined as milk collected between December–March and summer/outdoor period as milk collected between June and October. For the purposes of this analysis, skimmed, semi-skimmed and whole milk samples constitute the pasteurised milk samples (*n* = 36) and unpasteurised samples (*n* = 12) were those collected pre-pasteurisation and pre-homogenisation.

### 2.2. Milk Digestion

To extract iodine and selenium prior to analysis, milk samples were digested. For iodine determination, 0.5 mL of milk was added to 5 mL of 5% tetramethylammonium hydroxide (TMAH) (>99%, Acros Organics, Loughborough, UK). Samples were placed in the oven at 90 °C for 3 h. Samples were removed from the oven to cool and 0.5 mL of 1300 ng/g tellurium, an internal standard (Sigma Aldrich, Dorset, UK) in 1% TMAH was added and samples were made up to 50 g with 1% TMAH. For selenium determination, 3 g of milk was added to 1 mL high purity water and 6 mL concentrated nitric acid (VWR International, Leicestershire, UK). Samples were pre-heated for 10 min on a thermal plate before being placed in a Transform 680 Microwave Digestion System (Aurora, Vancover, BC, Canada). Following digestion, samples were cooled and 1.5 mL of 30% hydrogen peroxide added (Sigma Aldrich, Dorset, UK). Samples were made up to 50 g with high-purity water. 

### 2.3. Iodine and Selenium Analysis of Milk

The milk digests were shipped to LGC, Teddington, Middlesex, UK, for analysis. The iodine and selenium concentrations of milk were determined by inductively coupled plasma-mass spectrometry (ICP-MS) with 8800 ICP-QQQ-MS (Triple Quadrupole ICP-MS) (Agilent Technologies, Cheshire, UK). For iodine determination the instrument was operated in standard single quadrupole mode as iodine and tellurium do not suffer from interferences. All glassware (nebuliser, spray chamber and injector) were soaked overnight in 5% TMAH and rinsed with ultrapure water. The torch position, sample gas flow and lenses were optimised to ensure maximum signal intensity. For selenium analysis the instrument was operated in tandem mass spectrometry (MS/MS) mode using the H_2_ gas mode to effectively remove possible interferences on selenium. 

To ensure maximum accuracy, external calibration was undertaken using a stock standard, prepared gravimetrically from high purity potassium iodide (>99.99%, Alfa Aesar, Leicestershire, UK) dissolved in 5% TMAH (Sigma Aldrich, Dorset, UK) for iodine determination and for selenium analysis a stock standard containing 995 μg/g Se in 5% HNO_3_ (VHG Labs, Manchester, NH, USA) was used to prepare calibration standards. The uncertainty of the method for selenium analysis was calculated as ±10% using International Organisation for Standardisation (ISO) methodology [38] and was calculated to be ±15% for iodine [38]. Certified reference materials (CRM) were used to expose any systemic errors in the analysis. For this analysis ERM-BD150 (LGC Standards, Teddington, UK) was used. The certified value for selenium was 0.19 mg/kg and for iodine was 1.78 mg/kg. For each analysis 3 samples of the CRM were measured. The mean selenium value for the CRM was 0.18 mg/kg, a recovery of 96.5%. The mean iodine value for the CRM was 1.92 mg/kg, a recovery of 107.9%. The limit of detection (LOD) and limit of quantification (LOQ) for the analysis of milk iodine concentrations were 4.96 μg/L and 19.85 μg/L respectively. The LOD and LOQ for the analysis of milk selenium concentrations were 1.33 μg/L and 3.83 μg/L respectively.

### 2.4. Population Dietary Data

Data was obtained from the National Diet and Nutrition Survey (NDNS) Rolling Programme Years 1–4 (2008/2009–2011/2012) which provided nationally representative data on typical milk consumption (g/day) and iodine and selenium dietary intakes (µg/day) for the UK population [39]. Dietary data were collected for a total of 16,539 diary days from a total of 4156 participants who provided three or four day estimated weight food diaries [40]. 

The mean (12 months) iodine and selenium values for pasteurised (skimmed, semi-skimmed and whole) NI milk as measured in this study were applied to current NDNS milk consumption data. From this the daily iodine and selenium intakes from milk were simulated and comparisons were made between estimated intakes using NI milk data and estimated intakes as reported in the NDNS [21].

The theoretical impact of consuming NI milk on iodine and selenium intakes was evaluated for the entire NDNS population and by age group (1.5–3 years; 4–6 years; 7–10 years; 11–14 years; 15–17 years; 18–45 years; >46 years). Particular focus was given to groups thought to be most prone to low iodine intakes (girls aged 11–17 years and women of childbearing age 18–45 years) [10,12]. Following the dietary modelling, the estimated total daily iodine and selenium intakes (as estimated in NDNS using Food Standards Agency (FSA) (2002) data [23] or data from the current study) were compared to age and/or sex-specific dietary reference values for iodine and selenium (including the lower reference nutrient intake (LRNI), reference nutrient intake (RNI) and tolerable upper limit (TUL)) [41,42].

### 2.5. Statistical Analysis

Statistical analysis was conducted using Statistical Package for Social Sciences (SPSS) version 22 (International Business Machines (IBM), Chicago, IL, USA). Normality was assessed using the Shapiro-Wilks test. Results for non-parametric data are presented as median (25th–75th percentile). Results for parametric data are presented as mean and standard deviation (SD). A *p*-value of <0.05 was considered statistically significant throughout. One-way analysis of variance (ANOVA) (with Bonferroni correction for post hoc analysis) were used to assess any differences in the iodine and selenium concentration between types of milk and season. Independent t-tests were used to compare total milk intake (g/day) between children (1.5–17 years) and adults (≥18 years), and between males and females. Wilcoxon Signed Rank Tests were used to compare differences in iodine and selenium intake as estimated in NDNS using FSA (2002) data [23] or data from the current study. To compare the differences in theoretical iodine intakes from milk across seasons using the concentrations measured in the current study, the Friedman test was conducted.

## 3. Results

### 3.1. Iodine and Selenium Concentrations of Milk

**Figure 1:**
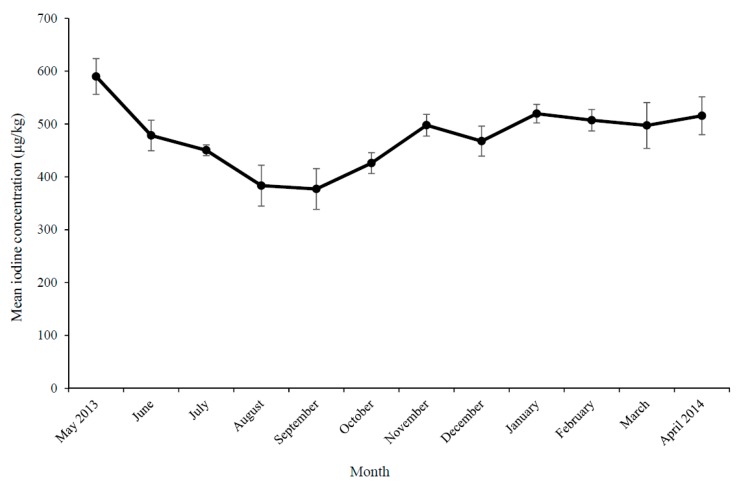
(Mean ± standard deviation (SD)) Iodine concentration of Northern Ireland (NI) milk (combined skimmed, semi-skimmed and whole) according to month of collection.

**Figure 2:**
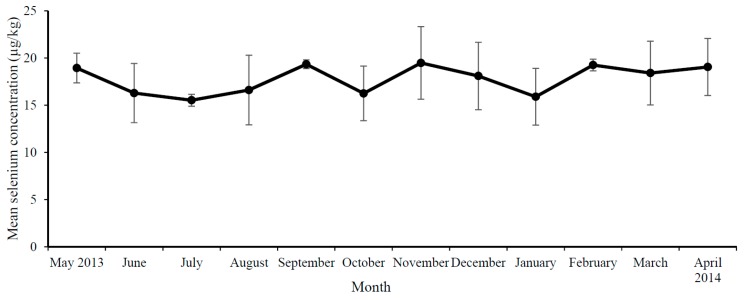
(Mean ± standard deviation (SD)) Selenium concentration of NI milk (combined skimmed, semi-skimmed and whole) according to month of collection.

In total, 12 monthly samples of each milk type (skimmed, semi-skimmed, whole and unpasteurised milk) were analysed. The year-round mean ± SD iodine and selenium concentration of pasteurised NI milk was 475.9 ± 63.5 µg/kg and 17.8 ± 2.7 µg/kg, respectively. Figure 1 shows the iodine concentrations and Figure 2 shows the selenium concentrations of NI milk (combined, skimmed, semi-skimmed and whole) according to month of collection. A significant difference was apparent between seasons for iodine only, with the lowest milk iodine concentration in spring (March–May) and highest in autumn (September–November) (Table 1). When milk samples were categorised as winter/indoor (December–March) or summer/outdoor (June–October), milk iodine concentration was significantly higher in winter/indoor period than summer/outdoor milk (Table 1). There were no significant differences in milk selenium concentration between winter/indoor or summer/outdoor milk. There were no significant differences in iodine or selenium concentration between milk fat class or between pasteurised and unpasteurised milk (Table 1).

### 3.2. UK Population Milk Intakes

Dietary intake data collected as part of the NDNS were evaluated for milk consumption and iodine and selenium intakes. For the entire NDNS population, milk was consumed on 84.4% of food diary days. For the entire NDNS population, daily milk intake was estimated to be 186.1 ± 169.6 g/day. Semi-skimmed milk was the most commonly consumed milk type (53.1% of diary days), whilst whole milk and skimmed milk were consumed on 22.4% and 8.1% of diary days respectively. Whole milk was consumed in the largest quantities (246.4 ± 207.2 g/eating occasion), while the mean consumption of semi-skimmed milk was 188.3 ± 173.6 g/eating occasion and for skimmed milk was 165.9 ± 171.1 g/eating occasion. Children (1.5–17 years) had a significantly higher daily milk intake than adults (≥18 years) (215.0 ± 196.2 vs. 159.8 ± 135.9 g/day; *p* < 0.001). For the entire NDNS population, males had a significantly higher daily milk intake in comparison to females (210.2 ± 192.4 vs. 164.5 ± 142.7 g/day; *p* < 0.001).

### 3.3. Theoretical Influence of Milk Consumption and Season on Iodine and Selenium Intakes

The contribution of milk to iodine and selenium intakes for all age groups is shown in Table 2 and Table 3, respectively. For both nutrients, estimates based on FSA (2002) data [23] were compared to the milk iodine and selenium concentrations measured in the present study for NI milk. Estimated daily iodine intakes from milk were significantly higher when the milk concentrations measured in the present study were applied to milk intake data in comparison to NDNS estimates (median (IQR) 66.2 (19.0–127.5) µg/day vs. 42.5 (12.1–81.3) µg/day; *p* < 0.001). Similarly, selenium intakes from milk were also significantly increased when milk selenium concentrations measured in the present study were applied to milk intake data and compared with NDNS estimates (2.5 (0.7–4.8) µg/day vs. 1.4 (0.4–2.7) µg/day; *p* < 0.001).

Using FSA (2002) milk iodine and selenium concentrations, daily iodine intake was 134.4 µg/day and daily selenium intake was 37.0 µg/day. When the updated NI milk iodine and selenium concentrations were applied, daily iodine intake was 165.0 µg/day and daily selenium intake was 39.0 µg/day. Using FSA (2002) milk iodine and selenium concentrations, milk provided 44.0 µg of iodine per day and 1.5 µg of selenium per day for NDNS participants. When the updated NI milk iodine and selenium concentrations were applied to milk intake data, milk provided 69.0 µg of iodine/day and 2.6 µg of selenium/day for NDNS participants. 

As outlined in Table 2 and Table 3, when the milk iodine and selenium concentrations were manipulated to values measured as part of the present study a higher proportion of NDNS participants met their respective iodine and selenium reference nutrient intakes (RNI). 

Applying milk iodine concentrations for each season as measured in the present study to milk consumption data, there was a significant difference in daily iodine intake from milk across seasons (*p* < 0.001). Milk from the spring months (March–May) provided the greatest contribution to iodine intake (77.5 (38.1–136.2) µg/day) while autumn milk (September–November) provided the smallest contribution to daily iodine intake (62.9 (30.9–110.6) µg/day). The median contribution of winter milk to iodine intakes was 72.2 (35.5–127) µg/day while summer milk was estimated to contribute 63.4 (31.2–111.5) µg/day. 

## 4. Discussion

This study has provided a comprehensive analysis of the iodine and selenium concentrations of cow’s milk produced in NI across the year. The year-round mean iodine concentrations in the present study (476 µg/kg) was higher than previously reported in UK food composition databases [43] and higher than those found in earlier studies from 1985 to 1994 [44]. These increases in milk iodine concentrations since the 1970s are not unique to the UK, but have also been reported in other European countries [18,30]. When the year-round milk iodine concentrations measured in the present study are compared to those reported by Wenlock et al. in 1982 [45], there has been a 246 µg/kg (107%) increase in milk iodine concentrations over the last 30 years. The selenium concentrations in milk have been less well monitored and therefore the data available are limited. Higher milk selenium concentrations have been measured in the present study in comparison to those listed in UK food composition databases [43], while two other studies have reported similar milk selenium concentrations to those reported in the present study [29,46]. The increase in milk iodine concentrations from those reported in the 1970s may be owing, at least in part, to the addition of iodine to animal feed and residues from the use of iodophors which are used to sterilise cows’ teats and milking vessels [36,47,48]. 

Although it appears that milk iodine and selenium concentrations have increased since the 1970s [44,49], it is important to note that the results of individual studies are not directly comparable with results of the present study as sampling has not been from the same dairies or geographical location. In addition, different analytical methods have been used to analyse milk and the sensitivity of analysis has improved over time. As the milk samples for the present analysis were collected in NI, a region in close proximity to the coast with high levels of iodine and selenium in the soil [50,51] it is likely that forages in NI are high in iodine and selenium. This natural enrichment may help explain the fact that although there was a significant difference in milk iodine concentrations between winter/indoor and summer/outdoor milk, the difference was not as pronounced as expected and no difference was observed for selenium concentrations. More careful monitoring and further exploration of the factors affecting the iodine and selenium concentrations in milk across the UK is warranted, particularly concerning the use of nutritional supplements such as seaweeds. 

Within NI, dairy production systems typically house cattle indoors in winter while in summer cattle are on pasture [52]. Winter milk has been reported to have a significantly higher iodine and selenium concentration than summer milk and this has been attributed to the mineral supplemented feed provided to cattle [29,30]. A seasonal variation in milk iodine concentration has been observed in this study where concentrations were highest in spring and lowest in autumn (534 vs. 434 µg/kg). These seasonal differences in milk iodine concentrations are not generalizable across dairy production systems. Seasonal differences in milk iodine concentrations would not be expected in dairy production systems that are based on fully housed or zero grazing practices which enable year round micronutrient supplementation. In countries with a moderate climate such as the UK and Ireland, calving tends to occur in winter-spring (February–April). Such husbandry means that milk yield is higher in the summer-autumn months and the concentration of trace elements in milk tends to be lower [53]. In both NI and the Republic of Ireland, more than 80% of dairy farms operate a spring calving season [54] and as research suggests iodine concentrations are lower in milk produced at later stages of lactation [55]; this may in part explain our observation that milk iodine concentrations were highest in spring. Nutrition during the dry period (pre-calving) may also have implications for milk iodine concentrations. A recent study of Irish dairy farms, reported that >95% of farmers fed pre-calving minerals to cows during the dry period and of this, 57% fed minerals for 6–8 weeks pre-calving [56]. Pre-calving minerals often contain high amounts of iodine and selenium which may increase the concentrations of these minerals in milk produced during early lactation [57]. Whilst there were differences in the milk iodine concentrations between winter and summer milk, the difference did not reach statistical significance contrary to the findings of many other studies [18,30,37,53]. In the present study, no seasonal differences in milk selenium concentrations were observed, conflicting with previous research [29]. This result was unexpected and went against our hypothesis, given that selenium is also sourced primarily from animal feed. Fordyce et al. (2009) analysed milk samples produced in Scotland and reported the selenium concentration of winter milk to be 21.7 µg/kg while selenium concentrations of summer milk were 17.5 µg/kg [29]. Further research is required to understand the factors that contribute to iodine and selenium fluctuations in milk, including the amount of supplemental feed offered throughout the year. In order to confidently assess seasonal differences for selenium concentrations a larger sample size may be required as the concentrations to be detected are low relative to the limit of quantification for this particular analytical method. 

Our results confirm those of previous work which reports that milk fat class does not affect the iodine or selenium concentration of milk [34,44,46]. The current study also found no significant effect of pasteurisation on the iodine or selenium concentrations of milk. Given the low consumption of unpasteurised milk in the UK (0.01% of total milk consumption) [58], pasteurisation of milk is unlikely to significantly influence iodine and selenium intake, and thus status. It is important, however, to acknowledge that the sample numbers in the current study were small and unbalanced for this analysis (12 unpasteurised and 36 pasteurised milk samples). To fully evaluate the differences between pasteurised and unpasteurised milk samples, a larger, balanced study is required. 

Iodine deficiency is a concern in women of childbearing age in the UK [10,12] and in the absence of an iodised salt programme, investigation of alternative dietary strategies to improve iodine intake and prevent iodine deficiency is therefore required. Using a dietary modelling approach, the current paper confirms that milk makes an important contribution to iodine intake and also contributes to dietary selenium intakes. This contribution is even greater when the milk iodine and selenium concentrations measured in this study are applied to population milk intake data. For the entire NDNS population, daily milk intake was estimated to be 186.1 g/day. When the milk iodine and selenium concentrations measured in this study were applied to milk intake data, milk provided a greater contribution to dietary intakes than estimates made using FSA (2002) data [23]. This highlights the need for a more widespread update and regular monitoring of UK milk iodine concentrations as we could be inaccurately estimating iodine and selenium intakes. This is particularly important for estimating iodine intake within vulnerable population groups. This dietary modelling scenario demonstrated the efficacy of NI milk, at increasing iodine intake, and reducing the proportion of the population failing to meet nutrient intakes. This finding is particularly promising for those population groups most vulnerable to iodine deficiency, such as adolescent females and women of childbearing age. It is noteworthy that although iodine intake from milk may be higher than estimated using food composition data, iodine deficiency remains a problem in the UK and Ireland, particularly for women of childbearing age and adolescent girls [9,10,11,12]. NDNS results show that other dairy products, namely cheese, yoghurt and cream contribute to iodine and selenium intakes, albeit the contribution is considerably smaller than from milk [12]. Given the increased milk iodine and selenium concentrations reported presently, the iodine and selenium concentrations in other dairy products and the contribution of these products to iodine and selenium intakes should be considered in future research. 

This study is not without its limitations. Firstly, the number of milk samples analysed was relatively small and the effect of pasteurisation on milk iodine and selenium concentrations should be confirmed in a larger, more representative study. Previous research has demonstrated that organic milk has a significantly lower iodine concentration than conventional milk [34,35], however, it was not possible to account for the consumption of organic milk in the present analysis as this data is not collected as part of the NDNS. There may have been differences in the trace element concentration of milk from the two different creameries which were not captured in this study with samples being combined. However, although this could be considered a limitation of the present study, this is how data are compiled for food composition tables. Although the milk analysis is specific to NI, this study provides an interesting insight to the potential effect of increased milk iodine and selenium concentrations, as observed in other European countries, on dietary nutrient intake. In this analysis it was not possible to link seasonal fluctuations in milk iodine and selenium concentrations with farm level data, which is an important consideration for future studies to assess. Future research should quantify the effect of milk consumption at current iodine and selenium levels on biomarkers of nutrient status.

## 5. Conclusions

The measured iodine and selenium concentrations of NI milk were higher than expected based on a comparison to the most recent UK estimates for milk [23,43]. This finding suggests that frequent monitoring of milk samples produced across the UK is warranted to allow more accurate estimates of the contributions of milk to nutrient intakes to be assessed. The dietary modelling approach taken in this study confirmed that milk makes an important contribution to iodine and selenium intakes, and this contribution may be higher than previously estimated if iodine and selenium concentrations measured in the present study were replicable across the whole of the UK at the current level of milk consumption. Iodine intakes were theoretically shown to vary by season concurrent with the seasonal variation in NI milk iodine concentrations. Routine monitoring of milk iodine concentrations is important and efforts should be made to understand reasons for fluctuations in milk iodine concentrations, in order to realise the nutritional impact to consumers. This is particularly important for iodine, as deficiency remains a public health issue in the UK and Ireland.

## Figures and Tables

**Table 1:** Iodine and selenium concentrations according to milk fat class, pasteurisation and season.

		*n*	Mean Iodine Concentration (µg/kg)	SD	*p* *	Mean Selenium Concentration (µg/kg)	SD	*p* *
Milk Fat Class	Skimmed	12	472.6	76.0	0.696	18.5	2.0	0.539
	Semi-Skimmed	12	466.6	62.6		17.6	3.3	
	Whole	12	488.5	53.3		17.2	2.8	
Pasteurisation	Pasteurised ^†^	36	475.9	63.5	0.275	17.8	2.7	0.438
	Unpasteurised ^‡^	12	451.7	71.9		18.4	2.0	
Season ^†^	Winter	9	498.1 ^a,b^	30.6	<0.001	17.8	2.8	0.180
	Spring	9	534.3 ^a^	53.7		18.8	2.4	
	Summer	9	437.4 ^b,c^	48.9		16.1	2.5	
	Autumn	9	433.6 ^c^	57.8		18.4	2.9	
Season/Housing	Winter/Indoor	12	497.9	47.5	<0.001	17.9	2.8	0.525
	Summer/Outdoor	15	423.1	47.2		16.8	2.6	

*n =* number of samples; SD = standard deviation; * *p* value for comparison between groups from one-way analysis of variance (ANOVA); ^†^ Mean of skimmed, semi-skimmed and whole milk samples; ^‡^ Samples were collected pre-pasteurisation and pre-homogenisation; ^a,b,c^ values within a column with different superscript letters represent statistical significance between groups (*p* < 0.05).

**Table 2:** Estimated iodine intake using National Diet and Nutrition Survey (NDNS) milk consumption data at iodine concentrations reported by the Food Standards Agency (2002) [23] and those measured in the present study of NI milk.

		Milk Consumption (g/day)		Estimated Iodine Intake (Food Standards Agency, 2002)								Theoretical Iodine Intake (NI Current Study)							
				From Milk (300 µg/kg) (µg/day)		From Total Diet (µg/day)		% Contribution of Milk to Total Intake	<LRNI (%)	Meeting RNI (%)	>TUL (%)	From Milk (475.9 µg/kg) (µg/day)		From Total Diet (µg/day)		% Contribution of Milk to Total Intake	<LRNI (%)	Meeting RNI (%)	>TUL (%)
1.5–3	386	297.6	155.8–460.2	90.3	47.3–139.6	128.7	96.4–184.4	70.2	0.8	71.8	18.9	141.6	74.1–219.0	183.9	130.2–265.4	77.0	0.8	52.1	42.7
4–6	361	206.3	112.5–337.5	62.6	34.1–102.4	127.8	94.1–171.8	49.0	3.0	65.4	4.7	98.2	53.4–160.6	163.2	119.8–227.3	60.2	2.8	65.9	17.2
7–10	442	168.8	98.3–269.3	51.2	29.8–81.7	122.9	94.1–170.8	41.7	4.3	57.9	2.7	80.3	46.8–128.2	154.0	110.7–212.8	52.1	2.7	67.4	8.4
11–14	440	125.0	48.9–230.0	39.9	14.8–69.8	113.7	82.7–157.4	35.1	11.4	38.9	0.2	59.5	23.3–109.5	135.9	94.8–197.0	43.8	8.0	53.9	0.7
15–17	353	92.9	37.5–157.1	28.2	11.4–47.6	103.9	77.3–151.3	27.1	17.6	28.9	0.6	44.2	17.9–74.7	120.3	89.9–181.2	36.7	13.0	36.3	1.4
18–45	1031	106.7	50.0–190.0	32.4	15.2–57.6	136.8	97.1–196.0	23.7	11.2	48.2	0.1	50.8	23.8–90.4	161.7	112.9–225.5	31.4	7.8	60.2	0.4
>46	1143	150.0	81.3–246.3	45.5	24.6–74.7	172.8	125.9–232.4	26.3	2.6	66.5	0.1	71.4	38.7–117.2	202.4	148.3–271.2	35.3	1.8	78.3	0.5

*n =* Number of participants, IQR = Interquartile range, LRNI = Lower reference nutrient intake, RNI = Reference nutrient intake, TUL = Tolerable upper limit; LRNI (µg/day): Age 1.5–3 years = 40; Age 4–6 years = 50; Age 7–10 years = 55; Age 11–14 years = 65; Age 15–17 years = 70; Age 18–45 years = 70; Age > 46 years = 70 [41]; RNI (µg/day): Age 1.5–3 years = 70; Age 4–6 years = 100; Age 7–10 years = 110; Age 11–14 years = 130; Age 15–17 years = 140; Age 18–45years = 140; Age > 46 years = 140 [41] ; TUL (µg/day): Age 1.5–3 years = 200; Age 4–6 years = 250; Age 7–10 years = 300; Age 11–14 years = 450; Age 15–17 years = 500; Age 18–45 years = 600; Age > 46 years = 600 [42].

**Table 3:** Estimated selenium intake using National Diet and Nutrition Survey (NDNS) milk consumption data at selenium concentrations reported by the Food. Standards Agency (2002) [23] and those measured in the present study of NI milk.

		Milk Consumption (g/day)		Estimated Selenium Intake (Food Standards Agency, 2002)								Theoretical Selenium Intake (NI Current Study)							
				From Milk (10 µg/kg) (µg/day)		From Total Diet (µg/day)		% Contribution of Milk to Total Intake	<LRNI (%)	Meeting RNI (%)	>TUL (%)	From Milk (17.8 µg/kg) (µg/day)		From Total Diet (µg/day)		% Contribution of Milk to Total Intake	<LRNI (%)	Meeting RNI (%)	>TUL (%)
Age (Years)	*n*	Median	IQR	Median	IQR	Median	IQR					Median	IQR	Median	IQR				
1.5–3	386	297.6	155.8–460.2	3.0	1.6–4.6	23.5	19.0–28.7	12.8	0.3	89.9	0.3	5.3	2.8–8.2	26.1	20.9–31.5	20.3	0.3	95.3	0.3
4–6	361	206.3	112.5–337.5	2.1	1.1–3.4	28.6	23.6–35.0	7.3	0.3	88.9	0.0	3.7	2.0–6.0	30.4	25.5–36.4	12.2	0.3	90.3	0.0
7–10	442	168.8	98.3–269.3	1.7	1.0–2.7	32.6	27.0–40.5	5.2	1.6	62.4	0.2	3.0	1.8–4.8	34.3	28.4–41.6	8.7	1.4	68.6	0.2
11–14	440	125.0	48.9–230.0	1.3	0.5–2.3	37.2	30.7–45.2	3.5	9.3	25.5	0.0	2.2	0.9–4.1	38.6	31.4–47.0	5.7	7.7	29.3	0.0
Males 15–17	174	114.4	53.8–244.5	1.1	0.5–2.4	44.2	34.3–56.1	2.5	36.8	9.2	0.0	2.0	1.1–4.6	45.8	35.6–57.4	4.4	35.1	9.8	0.0
Females 15–17	442	116.1	55.0–199.8	0.8	0.3–1.4	34.1	27.1–41.8	2.3	71.5	9.5	0.0	1.3	0.4–2.5	35.2	27.9–43.1	3.7	69.3	9.5	0.0
Males 18–45	503	161.3	90.0–270.0	1.2	0.6–2.0	49.8	40.5–63.6	2.4	24.4	11.3	0.0	2.1	1.0–3.6	50.9	41.1–65.0	4.1	22.2	11.8	0.0
Females 18–45	179	75.0	25.0–140.3	1.0	0.5–1.8	38.8	29.4–50.8	2.6	53.7	14.1	0.2	1.8	0.9–3.2	39.7	30.5–51.6	8.7	51.2	15.5	0.2
Males >46	589	101.0	50.0–179.5	1.6	0.9–2.7	50.4	37.7–64.7	3.2	29.2	14.7	0.4	2.9	1.6–4.8	52.0	39.5–66.1	5.6	26.0	15.1	0.4
Females >46	640	141.1	77.5–225.8	1.4	0.8–2.3	41.6	32.5–55.1	3.4	45.5	18.0	0.0	2.5	1.4–4.0	43.3	33.7–56.3	5.8	42.9	20.5	0.0

*n =* Number of participants, IQR = Interquartile range, LRNI = Lower reference nutrient intake, RNI = Reference nutrient intake, TUL = Tolerable upper limit; LRNI (µg/day): Age 1.5–3 years = 7; Age 4–6 years = 10; Age 7–10 years = 16; Age 11–14 years = 25; Age 15–17 years = 40; Age 18–45 years = 40; Age > 46 years = 40 [41]; RNI (µg/day): Age 1.5–3 years = 15; Age 4–6 years = 20; Age 7–10 years = 30; Age 11–14 years = 45; Males aged 15–17 years=70; Females aged 15–17 years = 60; Males aged > 18 years = 75, Females aged > 18 years = 60 [41]; TUL (µg/day): Age 1.5–3 years = 60; Age 4–6 years = 90; Age 7–10 years = 130; Age 11–14 years = 200; Age 15–17 years = 250; Age 18–45 years = 300; Age > 46 years = 300 [42].
